# Prophylactic Anakinra to Prevent Neurotoxicity After CAR T-Cell Therapy in Aggressive B-Cell Lymphomas: A Single-Center Real-World Experience

**DOI:** 10.3390/cancers18111787

**Published:** 2026-05-29

**Authors:** Tina Schmid, Inna Shaforostova, Ulrike Bacher, Katja Seipel, Marie-Noelle Kronig, Thomas Pabst

**Affiliations:** 1 Department of Medical Oncology, Inselspital, University of Bern, 3010 Bern, Switzerland; tina.schmid2@extern.insel.ch (T.S.); katja.seipel@unibe.ch (K.S.); marie-noelle.kronig@insel.ch (M.-N.K.); 2 Department of Hematology, Inselspital, University of Bern, 3010 Bern, Switzerland; veraulrike.bacher@insel.ch; 3 Department for Biomedical Research (DBMR), University of Bern, 3008 Bern, Switzerland

**Keywords:** anakinra, prophylactic use, neurotoxicity, CAR T-cell therapy, diffuse large B-cell lymphoma

## Abstract

**Background/Objectives**: Chimeric antigen receptor T-cell (CAR T) therapy is an effective treatment for relapsed/refractory (r/r) aggressive B-cell lymphomas; however, acute toxicities, such as immune effector cell-associated neurotoxicity syndrome (ICANS), remain common. Interleukin-1 (IL-1) has been implicated in the pathogenesis of ICANS, suggesting that prophylactic anakinra, an IL-1 receptor antagonist, might reduce its incidence or severity. **Methods**: We retrospectively analyzed 80 patients with B-cell lymphomas who received CD19-directed CAR T-cell therapy (tisagenlecleucel, axicabtagene ciloleucel, or lisocabtagene maraleucel) at the Bern University Hospital between April 2019 and June 2022. One cohort received prophylactic anakinra (100 mg subcutaneously on days 0 to +6 post-infusion), while the comparison cohort did not. **Results**: The incidence of ICANS was similar between groups (14 patients, 35%) with anakinra vs. 10 (25%) in the standard cohort (*p* = 0.464). Rates of grade 3 ICANS were also comparable (eight (20%) vs. seven (18%), *p* > 0.999). Among patients who developed ICANS, median hospitalization was numerically shorter with anakinra (27 vs. 40 days, *p* = 0.077). Anakinra did not impair CAR T-cell expansion and was well tolerated, with no treatment-related adverse events. Survival outcomes, including overall survival (OS) and progression-free survival (PFS), were similar between cohorts. **Conclusions**: In summary, prophylactic anakinra did not reduce the incidence or severity of ICANS in our analysis; however, it may be associated with shorter hospitalization in affected patients. Whether this reflects a direct therapeutic effect or improved overall toxicity management remains uncertain. Larger prospective studies are warranted to further clarify the role of anakinra for prophylactic treatment of ICANS following CAR T-cell therapy.

## Simple Summary

Neurotoxicity is a potentially life-threatening complication following chimeric antigen receptor (CAR) T-cell therapy in patients with relapsed/refractory (r/r) aggressive B-cell lymphomas. Previous studies have suggested a role of interleukin-1 (IL-1) in the pathogenesis of neurotoxicity, particularly immune effector cell-associated neurotoxicity syndrome (ICANS). The aim of this retrospective study was to evaluate the prophylactic use of the IL-1 receptor antagonist anakinra in reducing the incidence and severity of ICANS. We analyzed two cohorts of patients receiving CAR T-cell therapy for r/r B-cell lymphomas: one group received prophylactic anakinra, while the other did not. Although no significant difference was observed in the incidence or severity of neurotoxicity between the two groups, the duration of hospitalization was notably shorter in patients who developed ICANS and received anakinra. Whether this finding reflects a direct therapeutic effect or improved overall toxicity management remains unclear, and further investigation is warranted.

## 1. Introduction

Patients with aggressive B-cell lymphomas can be cured with a first-line R-CHOP-based treatment (rituximab, cyclophosphamide, doxorubicin, vincristine, prednisolone) in more than 60% of patients. However, up to 40% of patients are either refractory to this therapy or experience relapse [1,2,3]. In cases of relapse, most commonly occurring within 2 years, high-dose chemotherapy (HDCT) followed by autologous stem cell transplantation (ASCT) is the next curative treatment option [1]. For patients developing relapse after HDCT/ASCT, or those with an early relapse or primary-refractory DLBCL, therapeutic options are limited and the prognosis remains poor with a median overall survival of 6 months [4].

Chimeric antigen receptor T-cell (CAR T-cell) therapy revolutionized the treatment and the prognosis of relapsed/refractory B-cell-lymphoma. Various meta-analyses have demonstrated response rates from 61% to 72% in patients with r/r DLBCL receiving CAR T-cell treatment [5,6,7,8,9]. Currently, there are three CD19 CAR T-cell products approved in Switzerland, namely tisagenlecleucel (tisa-cel, Kymriah), axicabtagene ciloleucel (axi-cel, Yescarta) and lisocabtagene maraleucel (liso-cel, Breyanzi).

The promising response rates of CAR T-cell therapy have been accompanied by specific and severe side effects, such as cytokine release syndrome (CRS) and immune effector cell-associated neurotoxicity syndrome (ICANS). Managing these toxicities remains a major challenge in CAR T-cell therapy [8,10]. In a recent retrospective analysis of CD19 CAR T-cell toxicity, CRS was observed in 75% of patients and ICANS in 43% of patients, with 20% developing severe (≥grade 3) ICANS [8,11,12]. Higher-grade ICANS tends to occur more frequently with CAR T-cell products that use CD28 as the costimulatory domain, affecting up to 45% of patients [8,13,14].

The pathogenesis of ICANS is not fully understood. Mechanisms involved include the passive diffusion of cytokines, such as IL-6 and IL-15, into the central nervous system (CNS), endothelial activation with disruption of the blood–brain barrier, and migration of CAR T-cells into the CNS [15,16,17]. Furthermore, patients with neurotoxicity have a significantly higher number of circulating CAR T-cells [18,19].

The presentation of ICANS varies in severity and intensity, typically involving impairment of consciousness, confusion, aphasia, and changes in handwriting, and possibly progressing to epileptic seizures [14,20]. To better assess characteristic manifestations, the CAR T-cell-therapy-associated toxicity (CARTOX) Working Group developed a standardized screening tool, known as the CARTOX Score [21]. Including other domains of neurotoxicity, such as level of consciousness, presence of seizures and motor impairments, the American Society for Transplantation and Cellular Therapy (ASTCT) created a grading system for the severity of ICANS, the immune effector cell-associated encephalopathy (ICE) Score, which is currently widely used [22].

Given the substantial challenge posed by the development of ICANS, which may necessitate intensive care and can be life threatening, the development of therapeutic strategies to prevent or mitigate neurotoxicity is of critical importance [23,24].

Therapeutic recommendations for ICANS are based on the ASTCT’s grading system. Corticosteroids, which exert nonspecific anti-inflammatory effects, including suppression of CAR T-cell activity, remain the cornerstone of neurotoxicity management. The dosage and route of administration vary according to ICANS grade. In patients with high-grade ICANS, close monitoring is required, often including electroencephalography, neuroimaging, and admission to the intensive care unit when indicated. In most cases, neurotoxicity is fully reversible under steroid therapy. However, the impact of corticosteroids on CAR T-cell expansion remains incompletely understood, and some data suggest a negative effect on remission rates and progression-free survival [14,25,26,27,28,29]. Furthermore, evidence suggests that high-grade ICANS negatively affects impact on overall survival, highlighting the need for novel strategies to prevent or ameliorate this severe complication [20].

In various studies aiming to understand the pathophysiology of CAR T-cell mediated toxicity, the role of IL-1β and macrophages has been explored. Giavridis et al. suggested, in a mouse model, that blocking the IL-1 pathway can prevent CRS/ICANS-related mortality while preserving antitumor effects [30,31].

Anakinra is a selective IL-1-β inhibitor currently used in treating rheumatic diseases such as rheumatoid arthritis or gout. Given its potential relevance to ICANS pathogenesis and its ability to cross the blood–brain barrier, in contrast to tocilizumab, anakinra has been applied in patients with severe or corticosteroid-resistant ICANS. Furthermore, its potential role in neurotoxicity prophylaxis in patients receiving CAR T-cell therapy is gaining increasing attention [21,32,33].

Recent clinical trials have shown that prophylactic administration of anakinra after CAR T-cell therapy may reduce rates of high-grade ICANS, while not impacting the efficacy of CAR T-cells [34]. A phase II trial administering anakinra prophylactically demonstrated a reduced rate of severe ICANS to 9.7%, compared to a reported incidence of 30% in previous clinical trials [35]. Following that, we aimed to investigate prophylactic treatment with anakinra in patients with relapsed/refractory aggressive B-cell lymphomas treated at our center. We conducted a retrospective analysis, comparing PFS, OS, toxicities and hospitalization duration in two cohorts of 40 patients, with and without prophylactic anakinra (days 0 to +6 post-CAR T-cell infusion).

## 2. Materials and Methods

We conducted a single-center retrospective observational study, analyzing data from r/r aggressive B-cell lymphoma patients, including patients with the CNS involvement treated with CAR T-cell therapy at the Department of Medical Oncology, Bern University Hospital, Switzerland, between 15 April 2019 and 10 June 2022 [36]. Eighty patients were included in the analysis. Patients were subdivided into two groups: the first (standard treatment) group of 40 patients did not receive anakinra, whereas the second (experimental) group (40 patients) received subcutaneous anakinra at a dose of 100 mg once daily from day 0 to day +6 following CAR T-cell infusion. The dose of anakinra was selected based on data from previous prospective trials [34,35]. The primary endpoint of the study was the incidence of ICANS in patients receiving prophylactic treatment with anakinra compared to the standard group. Secondary endpoints included progression-free survival (PFS), overall survival (OS) and duration of hospitalization. Grading of CRS and ICANS was performed retrospectively and uniformly according to the ASTCT consensus criteria [22]. Neurotoxicity assessment was based on the CARTOX/ICE score as part of the ICANS grading system [18].

The study was approved by the local ethics committee in Bern, Switzerland (decision number 2024-00536; approval date 24 April 2024).

Survival curves were analyzed using Kaplan–Meier curves. OS was defined as the time between CAR T-cell reinfusion and either death or last follow-up. PFS was defined as the time between CAR T-cell reinfusion and any other event, whichever occurred first: relapse, death or loss of follow-up. Metric data such as age, laboratory results or time to relapse were first tested for normal distribution using the Shapiro–Wilk test. All continuous variables were found to be non-normally distributed. Consequently, unpaired, non-normally distributed, continuous variables were compared using the Mann–Whitney test. Nominal data, such as gender, occurrence of toxicity, relapse and death, were compared using Fisher’s exact test. All *p*-values below 0.05 were considered statistically significant. GraphPad Prism 8 was used for all statistical analyses and graphical representation. Written informed consent was obtained from all patients, and the study adhered to the principles of the Declaration of Helsinki.

## 3. Results

### 3.1. Baseline Patient and Disease Characteristics

Of the 80 patients, 31 patients (39%) were female and 49 (61%) were males. Median age at diagnosis was 62 years (range: 34–79). A total of 78 patients (98%) were initially diagnosed with DLBCL, 30 (38%) of whom had transformed DLBCL. Two patients (5%) were diagnosed with high-grade B-cell lymphoma (HGBL). A total of 40 (50%) patients had stage IV disease according to the Ann Arbor staging system [37]. The presence of B-symptoms at initial diagnosis was more frequent in the group treated without anakinra (five (12%) vs. 19 (48%), *p* = 0.001). Otherwise, patient characteristics were balanced between the two groups. Baseline patient characteristics are summarized in Table 1.

### 3.2. Patient Characteristics at Time of CAR T-Cell Therapy

Patient characteristics at the time of CAR T-cell therapy are summarized in Table 2. Median age was 69 years, and patients had received a median of two prior therapy lines. Most had progressive disease before infusion, and 55 patients (69%) required bridging chemotherapy. Significant differences were observed in the lower frequency of elevated LDH prior to lymphodepletion (20 (50%) patients in the anakinra group vs. 36 (90%) in the standard group, *p* = 0.0002) and in the CAR T-cell product used, as liso-cel was used only in patients without anakinra (0 vs. six (15%) patients, *p* < 0.001). No significant differences in disease burden or bridging therapy before CAR T-cell treatment were found between the two groups.

### 3.3. Safety

#### 3.3.1. Cytokine Release Syndrome (CRS)

A total of 50 (63%) patients received tisa-cel, 24 (30%) received axi-cel and six (7%) liso-cel, respectively, with no anakinra administered in the liso-cel group. Any-grade CRS was observed in 71 (89%) patients, most of whom presented with grade 1 (42 patients; 53%) or grade 2 (25 patients; 31%) after a median of 3 days (range 0–21) from CAR T-cell infusion. There was no significant difference in the incidence of CRS between the two groups, with 37 patients (46%) in the experimental and 34 patients (42%) in the standard group. Therapeutic interventions for CRS, including the median dose of tocilizumab, steroids and siltuximab, did not differ significantly between the two groups and are presented in Table 3.

#### 3.3.2. Immune-Effector Associated Neurotoxicity (ICANS)

In total, 24 (30%) patients developed any-grade ICANS, specifically 14 patients (35%) in the experimental group and 10 (25%) in the standard group (*p* = 0.464). Overall, no statistically significant differences were observed between the two groups with respect to the incidence or severity of neurotoxicity, including severe (grade ≥ 3) ICANS. The median lowest CARTOX Score was 2 (range 0–9) after a median of 10 days (range 0–33) in the group treated with anakinra, as compared to 0 (range 0–9) after a median of 5 days (range 1–15) in the standard group (*p* = 0.295). Admission to the intensive care unit was needed in 17 (21%) patients, of whom 14 (18%) presented with ICANS, while the remaining admissions were due to severe CRS (*p* > 0.999). Patients presenting with ICANS in the prophylactic anakinra group had a shorter length of stay, with a median of 27 days (range: 15–52) as compared to 40 days (range: 20–55) in the standard group (*p* = 0.077) (Table 3). Patients in the prophylactic anakinra group had significantly lower IL-6 peaks in peripheral blood compared to the standard group (median 841 pg/mL vs. 4128 pg/mL, *p* = 0.047). Serum ferritin levels were similar between the groups, although the peak occurred later with anakinra (median 11 vs. 8 days, *p* = 0.041). CRP levels and CAR T-cell expansion, measured by droplet digital PCR (ddPCR), showed no differences between the groups [38]. Importantly, CAR T-cell expansion was not diminished with anakinra (Table 3).

### 3.4. Response Rates and Survival Outcomes

**Figure 1:**
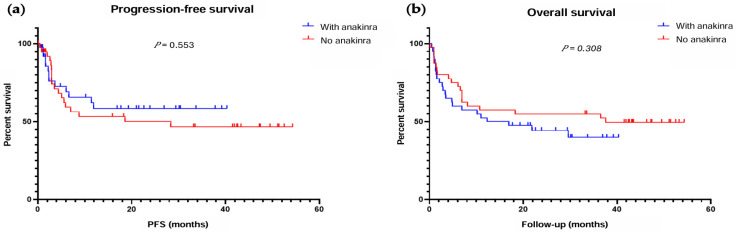
Survival after CAR T-cell therapy. (**a**) Progression-free survival; (**b**) overall survival.

Response rates and outcomes are summarized in Table 4 and shown in Figure 1. Median follow-up was 15 months (range 0.5–40) in the prophylactic anakinra group versus 35 months (range 0.1–54) in the standard group (*p* = 0.011). CR as best response was achieved in 19 (47%) patients of the experimental group versus 23 (59%) in the standard group (*p* = 0.502), and PR in 12 (33%) vs. eight (20%) (*p* = 0.210). Stable disease and progressive disease were similar between groups. At last follow-up, CR was observed in 16 (40%) patients of the experimental group and 21 (52%) of the standard group (*p* = 0.370), while PR was significantly higher in the experimental group (eight (20%) vs. one (3%), *p* = 0.029). Relapse occurred more frequently in the standard group (18 (45%) vs. 13 (33%)), although the difference was not statistically significant (*p* = 0.359). Mortality was comparable between the two groups (23 (58%) vs. 20 (50%) deaths, *p* = 0.654), with a median time to death of 4 months. Median OS was 15 months in the anakinra group versus 38 months in the standard group (*p* = 0.308). PFS was also similar between groups (Figure 1a,b). Median PFS was not reached in the anakinra group, with a 6-month PFS rate of 53% in both groups. Kaplan–Meier analyses of PFS and OS did not show statistically significant differences between the two groups.

## 4. Discussion

Previous studies have suggested that anakinra may be efficacious in preventing ICANS following CAR T-cell therapy; however, definitive data are still lacking. In this real-world, retrospective, single-center study, we evaluated the prophylactic use of anakinra in patients with relapsed or refractory aggressive B-cell lymphoma receiving CD19 CAR T-cell therapies. First, we failed to detect any significant differences in OS or PFS between patients who received prophylactic anakinra subcutaneously in the post-infusion period (days 0 to +6 after CAR T-cell infusion) and those who did not. Notably, the median follow-up was shorter in the anakinra group compared with the standard group, which may account for the lower observed relapse incidence. Six-month OS and PFS rates were comparable between the two groups, which is consistent with findings from prior studies [8,39]. Taken together, these results indicate that prophylactic anakinra is safe and does not adversely affect survival or relapse outcomes, although longer follow-up is required to confirm these observations.

At the last follow-up, the rate of partial remission was significantly higher in the anakinra group. This difference may be related to the shorter median follow-up in this cohort, as complete remission is frequently preceded by partial remission. No statistically significant differences in CR rates were observed between groups at any time point. Extended follow-up may clarify whether higher complete remission rates occur in the anakinra group.

Beyond OS and PFS, this study primarily focused on the assessment of neurotoxicity, which typically occurs within 4–7 days post-infusion, allowing for direct cohort comparison. Previous studies showed a significant reduction in high-grade (≥3) ICANS among patients receiving prophylactic anakinra, with rates of 10% and 9.7% compared with rates of up to 30% in prior clinical trials [8,34,35]. In contrast, we found no significant difference in any grade of ICANS between the two groups in our study. This discrepancy may be attributed to differences in dosing regimens, particularly the reactive dosage escalation in the case of clinical signs of CRS or ICANS in the study of Nath et al. and Park et al., whereas in our study, all patients in the anakinra group received a fixed dose from day 0 to day 6, independent of clinical presentation [34,35].

Interestingly, in our study, hospitalization duration for ICANS patients was shorter in the anakinra group compared with the standard group without anakinra (median 27 versus 40 days), though whether this results from anakinra use or improved toxicity management remains unclear. Larger, ideally prospective studies are needed to confirm this observation. Notably, the hospitalization duration was generally longer than in reported studies, possibly due to a high disease burden in most patients and the early era of CAR T-cell therapy when standardized management strategies were not yet established [40,41].

Importantly, anakinra did not affect CAR T-cell peak expansion in the peripheral blood measured by ddPCR in our patients, though its impact on long-term persistence was not assessed [42,43]. Encouragingly, no negative effects of anakinra were observed in this cohort, as response rates and survival were similar in both groups, and no adverse events were directly attributed to its use.

In this context, it is important to consider the current landscape of ICANS prevention. Currently, there are no standard preventive strategies for ICANS in patients receiving CAR T-cell therapies. The management of ICANS focuses on careful supportive care and early administration of corticosteroids for grade ≥2 neurotoxicity. Some other cytokine-targeted agents, such as emapalumab (IFN-γ-blockade), siltuximab (IL-6 blockade) and lenzilumab (GM-CSF blockade), have been investigated for prevention of ICANS, but robust clinical evidence supporting their prophylactic use is lacking [44]. Engineering-based strategies are advancing, with modifications to CAR design (e.g., altering costimulatory domains, affinity tuning) shown to reduce neurotoxicity in clinical trials without compromising efficacy; however, these approaches are not yet applicable in routine clinical practice [45].

Biomarker-guided approaches are being developed to stratify patients by neurotoxicity risk using clinical predictors (e.g., early CRS, inflammatory cytokine profiles), enabling tailored monitoring and preemptive interventions [46]. To summarize, careful supportive care and corticosteroids remain the cornerstone of early intervention for ICANS in the absence of established preventive treatment.

Our study has some limitations. First, as it is a retrospective analysis, there is selection bias as the two cohorts were enrolled consecutively, as well as a potential observer bias. Secondly, there were imbalances in baseline characteristics between the cohorts, including LDH levels and the presence of B-symptoms, which may have influenced toxicity, hospitalization, and survival outcomes. The follow-up period was relatively short, particularly in the anakinra group, which substantially limits direct comparisons between the cohorts. Patients treated in the anakinra group were enrolled more recently and may have benefited from improved risk management strategies for CAR T-cell products. In addition, the rate of CRS and hospitalization in our cohort was higher than previously reported, making interpretation more challenging. Furthermore, different CAR T-cell products with distinct toxicity profiles were used, further limiting the comparability and restricting the causal interpretation of the observed differences. Lastly, the sample size was small, reducing the statistical power of the study and limiting the ability to detect clinically significant differences in ICANS incidence between the two groups.

## 5. Conclusions

In conclusion, in this study, prophylactic anakinra administration did not demonstrate a reduction in the incidence of neurotoxicity, as outlined by the frequency of any-grade ICANS. However, its use was feasible and safe, with no observed impact on peak expansion of CAR T-cells and clinical outcomes regarding OS, PFS and remission rates. Nevertheless, we observed a shorter hospitalization duration in the group receiving anakinra compared to the standard group, which may be due to a low risk of high-grade CRS and ICANS, as well as more proactive toxicity management strategies. Therefore, our study, as well as the above reported studies, should encourage further prospective trials for a more comprehensive evaluation of this prophylactic approach.

## Figures and Tables

**Table 1:** Patient characteristics at time of first diagnosis.

Parameter	With Anakinra (%) (*n* = 40)	Without Anakinra (%) (*n* = 40)	*p*-Value
Female, *n* (%)	14 (35)	17 (43)	0.497
Male, *n* (%)	26 (65)	23 (57)	0.497
Age, years, median (range)	59 (36–79)	63 (34–77)	0.506
Initial Diagnosis, *n* (%)			
DLBCL	40 (100)	38 (95)	0.493
De novo DLBCL	28 (70)	20 (53)	0.109
Transformed DLBCL	12 (30)	18 (45)	0.248
HGBL	0	2 (5)	0.493
Transformed from FL	0	1 (3)	0.999
Stage at initial diagnosis, *n* (%)			
I	3 (7)	2 (5)	0.999
II	9 (23)	8 (20)	0.999
III	5 (12)	8 (20)	0.526
IV	19 (48)	21 (52)	0.823
Unknown	4 (10)	1 (3)	0.359
B-symptoms, % (*n*)	5 (12)	19 (48)	**0.001 ^a^**

DLBCL: diffuse large B-cell lymphoma; FL: follicular lymphoma; HGBL: high-grade B-cell lymphoma. ^a^ *p*-values < 0.05 are shown in bold.

**Table 2:** Patient characteristics at time of CAR T-cell therapy.

Parameter	With Anakinra (*n* = 40) (%)	Without Anakinra (*n* = 40) (%)	*p*-Value
Previous lines of therapy, median, (range)	2 (2–7)	2 (1–6)	0.496
Radiotherapy, *n* (%)	17 (43)	16 (40)	0.999
ASCT, *n* (%)	17 (43)	22 (55)	0.371
Diagnosis at time of CAR T-cell therapy, *n* (%)			
DLBCL	37 (94)	40 (100)	0.240
HGBL	1 (2)	0	0.999
PMBL	1 (2)	0	0.999
Burkitt-like lymphoma	1 (2)	0	0.999
Bridging chemotherapy, *n* (%)	21 (52)	23 (58)	0.822
Bridging radiotherapy, *n* (%)	8 (20)	4 (10)	0.348
Elevated LDH prior to LD, *n* (%)	20 (50)	36 (90)	**0.0002 ^a^**
Remission status prior to CAR T, *n* (%)			
CR	2 (5)	0	0.493
PR	10 (25)	7 (18)	0.585
SD	4 (10)	4 (10)	0.999
PD	24 (60)	29 (72)	0.344
CAR T-cell product, *n* (%)			**0.021 ^a^**
tisa-cel	25 (63)	25 (63)	0.999
axi-cel	15 (37)	9 (22)	0.222
liso-cel	0	6 (15)	**<0.001 ^a^**

ASCT: autologous stem cell transplant; axi-cel: axicabtagene ciloleucel; CR: complete remission; DLBCL: diffuse large B-cell lymphoma; HGBL: high-grade B-cell lymphoma; liso-cel: lisocabtagene maraleucel; LD: lymphodepletion; LDH: lactate dehydrogenase; PD: progressive disease; PMBL: primary mediastinal B-cell lymphoma; PR: partial remission; SD: stable disease; tisa-cel: tisagenlecleucel. ^a^ *p*-values < 0.05 are shown in bold.

**Table 3:** Toxicities and laboratory values.

Parameter	With Anakinra (*n* = 40) (%)	Without Anakinra (*n* = 40) (%)	*p*-Value
Grade of CRS *n* (%)			0.034
Total	37 (93)	34 (85)	0.481
1	27 (68)	15 (38)	0.013
2	9 (22)	16 (40)	0.147
3	1 (3)	1 (3)	0.999
4	0	2 (5)	0.493
Time to CRS, (days) (range)	3 (0–21)	3 (0–15)	0.942
Treatment *n* (%)			
Tocilizumab	30 (75)	21 (53)	0.061
G-CSF	38 (95)	22 (55)	0.002
Steroids	19 (48)	14 (35)	0.364
Siltuximab	5 (13)	6 (15)	0.999
ICANS total, *n* (%)	14 (35)	10 (25)	0.464
Grade 1	3 (8)	2 (5)	0.999
Grade 2	3 (8)	0	0.2405
Grade 3	8 (20)	7 (18)	0.999
Grade 4	0	1 (3)	0.999
CARTOX Score minimum, median (range)	2 (0–9)	0 (0–8)	0.295
CARTOX normalization time, days, median (range)	2 (1–12)	1 (1–10)	0.847
Transfer to the intensive care unit, *n* (%)	8 (20)	9 (23)	0.999
With ICANS	7 (18)	7 (18)	0.999
No ICANS	1 (3)	2 (5)	0.999
Duration of hospitalization, days, median (range)	22 (15–52)	21 (5–55)	0.510
With ICANS	27 (15–52)	40 (20–55)	0.077
No ICANS	21 (18–30)	20 (5–45)	0.276
Laboratory peak values, median (range)			
CRP [mg/L]	41 (3–272)	42 (3–271)	0.633
IL-6 [pg/mL]	687 (9–157,117)	329 (4–42,209)	0.523
Ferritin [pg/mL]	1187 (190–13,393)	1400 (99–12,398)	0.719
Expansion CAR T-cells [copies/µg DNA]	5085 (37–91,575)	5547 (54–218,384)	0.193

CARTOX: CAR T-cell-therapy-associated toxicity; CRP: C-reactive protein; CRS: cytokine release syndrome; DNA: deoxyribonucleic acid; G-CSF: granulocyte Colony-Stimulating Factor; ICANS: immune effector cell-associated neurotoxicity syndrome; IL-6: interleukin-6.

**Table 4:** Clinical outcomes after CAR T-cell therapy.

Parameter	With Anakinra (*n* = 40) (%)	Without Anakinra (*n* = 40) (%)	*p*-Value
Best remission status, *n* (%)			
CR	19 (47)	23 (59)	0.502
PR	13 (33)	8 (20)	0.210
SD	2 (5)	3 (7)	0.999
PD	6 (15)	6 (15)	0.999
Remission status at last follow-up, % (*n*)			0.509
CR	16 (40)	21 (52)	0.370
PR	8 (20)	1 (3)	0.029
SD	1 (3)	0	0.999
PD	15 (37)	18 (45)	0.650
Relapse, *n* (%)	13 (33)	18 (45)	0.359
Time to relapse, months, median, (range)	2.3 (0.4–12)	3 (0.1–28)	0.266
Death, *n* (%)	23 (58)	20 (50)	0.654
Time to death, months, median (range)	2.9 (0.5–30)	5.4 (0.1–38)	0.635
Median OS (months)	15	38	0.308
6-month OS (%)	63%	75%	
Median PFS (months)	not reached	28	0.553
6-month PFS (%)	53%	53%	
Follow-up, months, median (range)	15 (0.5–40)	35 (0.1–54)	**0.011 ^a^**

CR: complete remission; OS: overall survival; PD: progressive disease; PFS: progression-free survival; PR: partial remission; SD: stable disease. ^a^ *p*-values < 0.05 are shown in bold.

## Data Availability

The original contributions presented in this study are included in the article. Further inquiries can be directed to the corresponding authors.
